# Construction of Prediction Model for Atrial Fibrillation with Valvular Heart Disease Based on Machine Learning

**DOI:** 10.31083/j.rcm2307247

**Published:** 2022-06-28

**Authors:** Qiaoqiao Li, Shenghong Lei, Xueshan Luo, Jintao He, Yuan Fang, Hui Yang, Yang Liu, Chun-Yu Deng, Shulin Wu, Yu-Mei Xue, Fang Rao

**Affiliations:** ^1^Guangdong Cardiovascular Institute, Guangdong Provincial People’s Hospital, Guangdong Academy of Medical Sciences, 510080 Guangzhou, Guangdong, China; ^2^Research Center of Medical Sciences, Provincial Key Laboratory of Clinical Pharmacology, Guangdong Provincial People’s Hospital, Guangdong Academy of Medical Sciences, 510080 Guangzhou, Guangdong, China

**Keywords:** atrial fibrillation, valvular heart disease, WGCNA, machine leaning, specific markers

## Abstract

**Background:**

Valvular heart disease (VHD) is a major precipitating factor of atrial fibrillation (AF) that contributes to decreased cardiac function, heart failure, and stroke. Stroke induced by VHD combined with atrial fibrillation (AF-VHD) is a much more serious condition in comparison to VHD alone. The aim of this study was to explore the molecular mechanism governing VHD progression and to provide candidate treatment targets for AF-VHD.

**Methods:**

Four public mRNA microarray datasets were downloaded and differentially expressed genes (DEGs) screening was performed. Weighted gene correlation network analysis was carried out to detect key modules and explore their relationships and disease status. Candidate hub signature genes were then screened within the key module using machine learning methods. The receiver operating characteristic curve and nomogram model analysis were used to determine the potential clinical significance of the hub genes. Subsequently, target gene protein levels in independent human atrial tissue samples were detected using western blotting. Specific expression analysis of the hub genes in the tissue and cell samples was performed using single-cell sequencing analysis in the Human Protein Atlas tool.

**Results:**

A total of 819 common DEGs in combined datasets were screened. Fourteen modules were identified using the cut tree dynamic function. The cyan and purple modules were considered the most clinically significant for AF-VHD. Then, 25 hub genes in the cyan and purple modules were selected for further analysis. The pathways related to dilated cardiomyopathy, hypertrophic cardiomyopathy, and heart contraction were concentrated in the purple and cyan modules of the AF-VHD. Genes of importance (*CSRP3*, *MCOLN3*, *SLC25A5*, and *FIBP*) were then identified based on machine learning. Of these, *CSRP3* had a potential clinical significance and was specifically expressed in the heart tissue.

**Conclusions:**

The identified genes may play critical roles in the pathophysiological process of AF-VHD, providing new insights into VHD development to AF and helping to determine potential biomarkers and therapeutic targets for treating AF-VHD.

## 1. Introduction

Atrial fibrillation (AF) is the most prevalent arrhythmia within the general population [1]. Morbidity and mortality linked to AF represent a significant public health burden worldwide [2]. There are multiple factors contributing to AF, including valvular heart disease (VHD), hypertension, age, obesity, and diabetes [3, 4]. VHD is a significant cause of arrhythmia and stroke. Stroke induced by AF-VHD is a more serious condition compared to VHD alone [5, 6]. However, the mechanism for the development of VHD into AF-VHD is not yet fully understood. It is therefore essential to investigate the pathogenesis and clarify the precise molecular mechanisms involved in AF-VHD progression.

Lamirault *et al*. [7] identified the gene expression profile associated with human AF-VHD. In their study, ight atrial appendages in 11 patients with AF-VHD and 7 patients with sinus rhythm with VHD (SR-VHD) undergoing open heart surgery were included in cardiac-specific microarray analysis. The results indicated that 169 genes were differentially expressed between the two groups. Notably, cysteine- and glycine-rich protein 3 (*CSRP3*) was found to be highly expressed in AF-VHD and involved in cardiac contraction. Furthermore, Yan *et al*. [8] and Li *et al*. [9] screened key immune-related genes in AF-VHD. Liu *et al*. [10] also identified feature genes for AF with VHD using integrative transcriptomic, proteomic, and machine learning approaches. In our study, by contrast, we merged related datasets and used weighted gene co-expression network analysis (WGCNA), a statistical method that is similar to cluster analysis but is more biologically meaningful, and machine learning methods to identify specific biomarkers [11, 12]. Even though many studies have investigated AF-VHD markers, specific predictive biomarkers are still lacking to enable early detection. The communicative regulatory mechanisms of AF-VHD also remain poorly understood.

In the present study, co-expression networks were constructed using the dataset GSE115574’s expression profile to identify key modules and hub genes related to AF with VHD. The genes were then subjected to gene ontology (GO) and Kyoto Encyclopedia of Genes and Genomes (KEGG) ontology enrichment analyses. Subsequently, important genes (*CSRP3*, Transient Receptor Potential Channel Mucolipin 3 (*MCOLN3*), solute carrier family 25 member 5 (*SLC25A5*), and *FGF1* intracellular binding protein (*FIBP*)) were identified using a machine learning approach and their potential clinical significance was determined. Notably, the clinical significance of *CSRP3* and *MCOLN3* was statistically significant. Functional enrichment results showed that *CSRP3* has a strong association with heart development and *MCOLN3* is linked to the calcium channel complex. Additionally, the available literature indicated that *CSPR3* is closely involved in the process of cardiac hypertrophy [13, 14]. These observations may link AF with dilated or hypertrophic cardiomyopathy, providing novel evidence for the diagnosis and treatment of AF with VHD in a clinical setting.

## 2. Methods

### 2.1 Atrial Fibrillation Datasets Filtration

Gene Expression Ominibus (GEO, https://www.ncbi.nlm.nih.gov/geo/) was used to extract the raw datasets and included if datasets met the following criteria. (1) The expression profiling was acquired by array; (2) The experimental platform belonged to *GPL570* platform; (3) Data sets should include gene expression profiles of human left or right atria tissues; (4) All experiments included controls and the minimum sample size used was three.

### 2.2 Data Collection

Based on the criteria mentioned above, four publicly microarray datasets (GSE79768, GSE115574, GSE14975 and GSE41177) were filtered and downloaded. In the GSE115574 dataset, the expression matrix of a total of 59 samples was acquired from the human atrial tissue, containing 15 patients diagnosed with AF-VHD, and 15 with SR-VHD. Left atrial appendage and right atrial appendage samples (except 1 patient) were obtained from each patient. In GSE79768, a total of 7 patients with AF-VHD and 6 with SR-VHD provided atrial tissue samples. GSE14975 contained 5 atrial tissue samples from AF-VHD patients and 5 from SR-VHD control. The GSE41177 dataset included 16 patients with persistent AF receiving valvular surgery and 3 patients diagnosed with SR with VHD.

### 2.3 Data Preprocessing and Differentially Expressed Genes (DEGs) Screening

Furthermore, Probe IDs were mapped to gene symbols in each dataset by extracting them from the respective platform file. The microarray data preprocessing, containing normalization and background correction, was conducted by applying the “Affy” package in R [15]. Then, using the “sva” package of the R software (version 3.6.3) to merge and batch normalization four datasets. Afterwards, the “limma” package was used to identify the DEGs between the AF-VHD and SR-VHD. The statistical cutoff values were an absolute $\log_{2}$ FC >0.3 (FC, fold change) and adjust *p*-value < 0.05 in combined datasets. Volcano plot and Heatmap were generated according to the data above by using R package “ggplot2” and “pheatmap”.

### 2.4 Functional Enrichment Analysis 

In order to explore the biological function of the DEGs and genes in key modules, GO analysis and a KEGG terms enrichment analysis were performed using Metascape tool (http://metascape.org) and ClusterProfiler (version 3.6.0) software in R language [16, 17]. Enrichment significance thresholds were set at an adjust *p*-value below 0.05. Furthermore, Gene set enrichment analysis (GSEA) was performed by clusterProfiler (R package) and GSEA plots were visualized by “gseaplot” package [18]. Results with a |NES|>1 and FDR <0.25 were regarded as statistically significant (NES, normalized enrichment score; FDR, false discovery rate).

### 2.5 Construction of Weighted Gene Co-Expression Network

Based on the median absolute deviation of the genes, we selected the top 5000 genes for WGCNA using the R package “WGCNA” [19, 20]. Biological networks were constructed with a value of 9 for the soft thresholding parameter to satisfy the scale-free assumption (linear regression model fitting index $R^{2}$ = 0.91). We calculated pairwise Pearson’s correlation matrix and then transformed it into an adjacency matrix. A Topological Overlap Measure (TOM)-based dissimilarity matrix (DissTOM) was created by transforming the adjacency matrix, and modules were generated by hierarchical average linkage clustering analysis for the gene dendrogram. After acquiring modules, module eigengene (ME), first principal component of the expression matrix of the referred to module, was calculated using the “Module Eigengenes” function. The relationship between clinical parameters and modules were indirectly assessed by looking at the correlation between MEs and clinical traits. Module significance (MS) was calculated by taking the average of the gene scores for all genes within a module. Modules with the highest MS scores were regarded as key modules and selected for subsequent analysis. Additionally, we extracted gene expression profiles of each module genes for further analysis.

### 2.6 Identification of Candidate Hub Genes in Key Module

The module membership (MM) was also calculated, which was regarded as the degree of association between the ME and the gene expression matrix. Then, the intramodular hub genes were identified based on gene significance (GS) >0.2 and MM >0.8 [21]. Heatmaps were conducted to demonstrate the putative candidate genes’ expression patterns with the R package “pheatmap” [22]. We also made Venn diagrams for common DEGs in four public microarray datasets and hub genes in WGCNA.

### 2.7 TF-miRNA Network of Hub Genes

The TF-miRNA regulatory network was constructed by overlapping TF-hub genes and miRNA-hub genes using the Network-Analyst database (http://www.networkanalyst.ca) and then visualized by performing Cytoscape (version 3.7.2) [23, 24, 25] (TF, transcription factor).

### 2.8 Machine Learning: Construction of Lasso and Random Forest Model

We performed least absolute shrinkage and selection operator (LASSO) regression by applying the “glmnet” package in R software to identify the candidate predictive features based on a generalized linear model [26]. Moreover, we constructed Random Forest (RF) Model to identify the important variables by using “Random Forest” package [27]. Finally, we screened real hub gene signatures by intersecting gene signatures from the LASSO and RF.

### 2.9 Development and Validation of a Prognostic Model

The association analysis between hub genes was performed by using the spearman rank correlation coefficient, illustrated by heat-map. To screen out the potential clinical significance of hub genes, the receiver operating characteristic curve (ROC) was created using “*pROC*” packages [28]. A multivariate regression formula was built based on the hub genes’ expression value and their regression coefficients under the merged datasets. Finally, a nomogram was constructed based on the selected predictive factors identified by using the “rms” package in R to predict the prevalence of AF-VHD. Calibration curves were plotted to evaluate the difference between the predicted probability and the actual probability. In addition, a decision curve analysis (DCA) can be used to measure the net benefit of a predictive model [29].

### 2.10 Patients

Left atrial appendages were obtained from 6 VHD patients with AF and 5 VHD patients with SR undergoing open heart surgery. Patients with hyperthyroidism, diabetes, hypertension and infectious diseases were excluded from our study. After the surgical operation, liquid nitrogen was immediately applied to the tissue specimens. Human participants in the studies were reviewed and approved by the ethics committee of the Guangdong General Hospital, Guangdong Academy of Medical Sciences (No. GDREC2017111H). A signed informed consent form was provided to all patients and their legal representatives.

### 2.11 Western Blot Analysis

The experiment was conducted based on the procedure reported as previously [30]. Antibodies used were mentioned as follows: anti-CSRP3 antibody [1:1000, Abcam Cat# ab172952]; anti-MCOLN3 antibody [1:1000, Thermo Fisher Invitrogen Cat# PA5-109339]; anti-GAPDH antibody [1:5000, Proteintech Cat# 60004-1-lg].

### 2.12 Specific Expression Analysis of Hub Genes in Tissue and Cells

We used Human Protein Atlas (HPA) (https://www.proteinatlas.org/) tool to validate the mRNA and protein expression levels of the hub genes in all tissues. The Single Cell Type Atlas part in HPA as used to illustrate the expression of hub genes in single specific cell types [31].

### 2.13 Statistical Analysis

A description of the bioinformatic analyses appeared in corresponding subsections. In all cases, values were expressed as means and standard deviations (SD), and Student’s *t*-test was used to determine pairwise statistical significance of the differences between two group means. A *p*-value < 0.05 was defined as statistically significant.

## 3. Results

### 3.1 Study Workflow

**Fig. 1.**
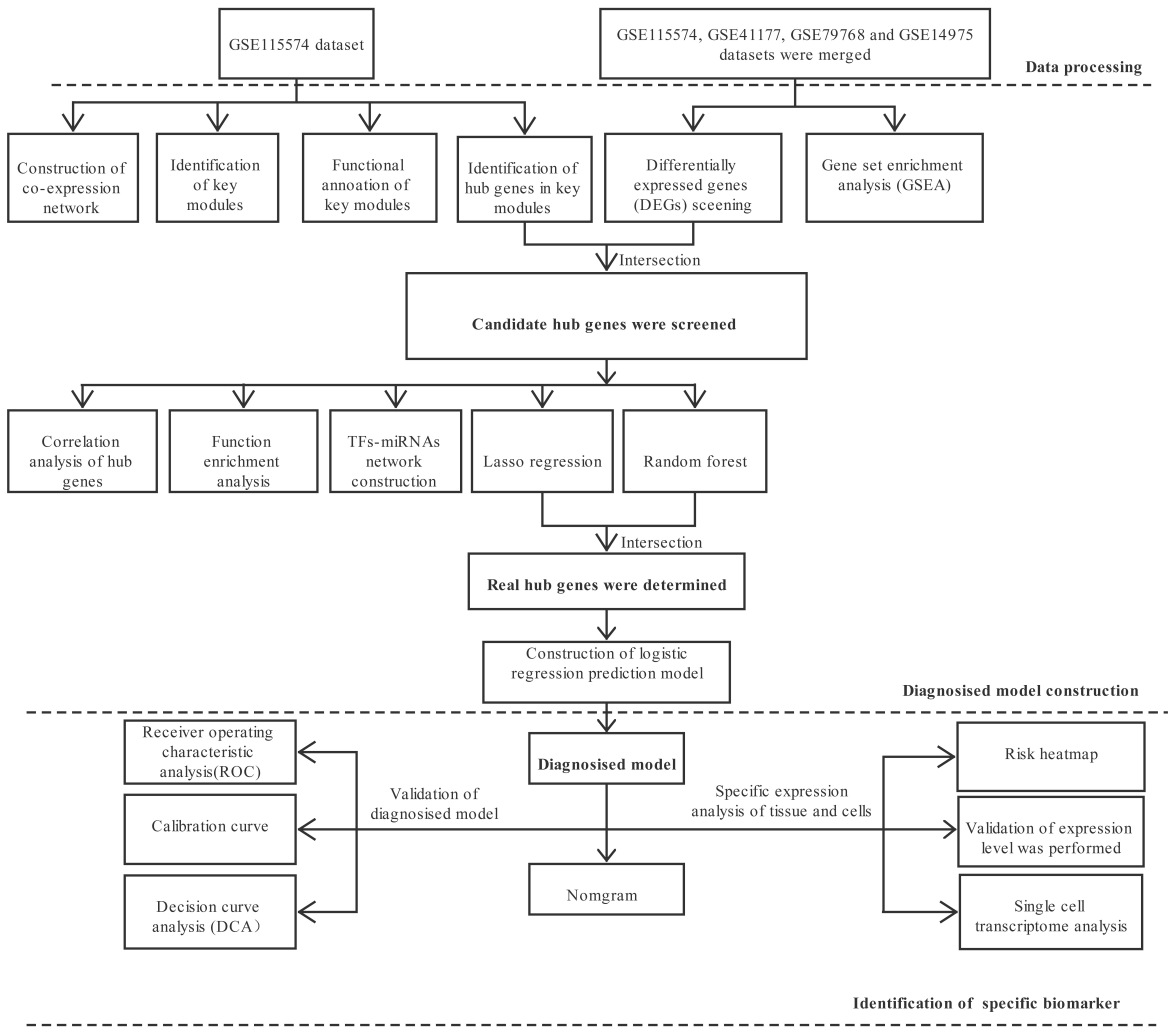
**Flowchart of the analysis strategy**.

The flowchart for the study analysis strategy is shown in Fig. 1. Fourteen modules of co-expressed genes were identified via weighted gene correlation network analysis. The cyan and purple modules were identified as the most clinically significant. GO and KEGG analyses were performed on both modules, with similar processes being carried out on the hub genes within the key modules. Crucial candidate genes were identified by intersecting the hub genes in WGCNA and common DEGs in merged datasets. TF-crucial genes and miRNA-crucial gene networks were visualized using Cytoscape. Machine learning methods, including Lasso and random forest, were implemented to select the potential significant genes and to construct a diagnosis prediction model. The ROC curve and DCA were utilized on this prediction model to assess the predictive power. Then, protein expression levels of important genes in AF-VHD were verified using western blot analysis. Specific expression analysis of hub genes in tissue and cell samples was performed to identify specific biomarkers.

### 3.2 DEGs Screening

**Fig. 2.**
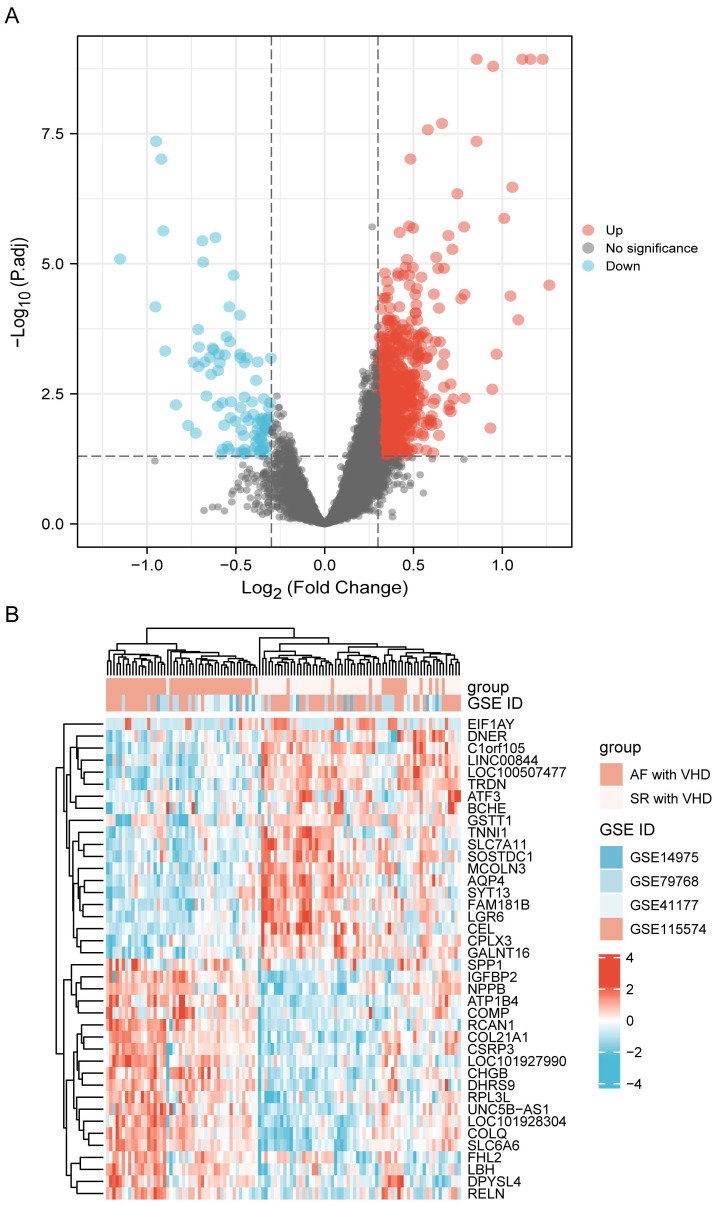
**DEG identification**. (A) Volcano plot visualization of differentially expressed genes and their statistical significance. The red dots indicate up-regulated genes, and the blue dots indicate down-regulated genes. (B) Heatmap showing expression profiles of top 40 DEGs. DEGs, differentially expressed genes.

Datasets GSE115574, GSE41177, GSE79768, and GSE14975 were included in the analysis. Based on the screening criteria, 819 genes in the merged datasets were screened out as common DEGs, of which 725 genes were up-regulated and 94 genes were down-regulated (Fig. 2A). DEGs were ranked according to the fold change expression, and the top 40 were represented using a heatmap (Fig. 2B).

### 3.3 Functional Enrichment of DEGs

**Fig. 3.**
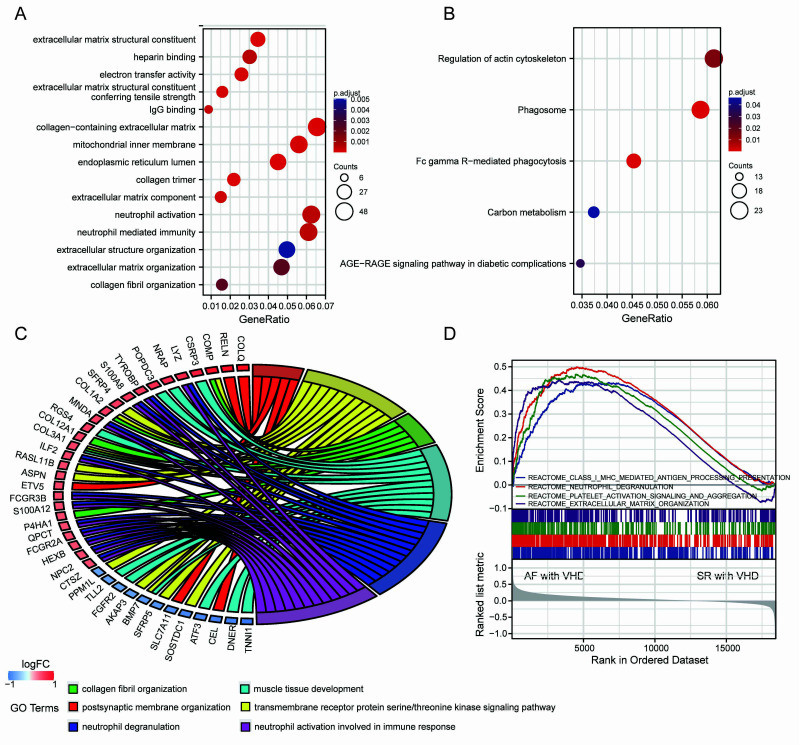
**Functional enrichment analysis of DEGs and GSEA results**. (A) GO term enrichment with top five significant adjusted *p*-values for DEGs illustrated in relation to biological process, molecular function, and cellular component. (B) Top five enriched KEGG pathways for DEGs. (C) Chord plots for gene enrichment analysis. (D) GSEA plots.

A ll DEGs were used in functional annotation analyses. The top five significant terms were displayed in bubble plots according to their adjusted *p*-values (Fig. 3A,B). The GO terms were associated with the molecular functions (MFs), cellular components (CCs), and biological processes (BPs). Those linked with MFs included extracellular matrix structural constituent, extracellular matrix structural conferring tensile strength, IgG binding, electron activity, and heparin binding. CCs included collagen-containing extracellular matrix, mitochondrial inner membrane, endoplasmic reticulum lumen, and collagen trimer. BPs included extracellular matrix organization, collagen fibril organization, neutrophil activation, and neutrophil-mediated immunity (Fig. 3A). Terms of the enriched KEGG pathway are represented in Fig. 3B, including the phagosome, Fc gamma R-mediated phagocytosis, regulation of actin cytoskeleton, carbon metabolism, and the advanced glycation end products (AGEs) and its synergetic receptor-AGEs-RAGE signaling pathway in diabetic complications. Detailed results are summarized in the **Supplementary Tables 1,2**. The top 6 BP enrichment terms were determined by their adjusted (adj) *p*-values and BgRatio values. and chord plots were used (Fig. 3C) in order to better understand the molecular functions of DEGs and the potential biological processes in which they could be involved. GSEA was performed based on all genes (Fig. 3D). The AF-VHD groups were enriched in terms of class I major histocompatibility complex (MHC)-mediated antigen presentation, neutrophil degranulation, platelet activation signaling and aggregation, and extracellular matrix organization.

### 3.4 Construction of Co-Expression Network Using WGCNA

**Fig. 4.**
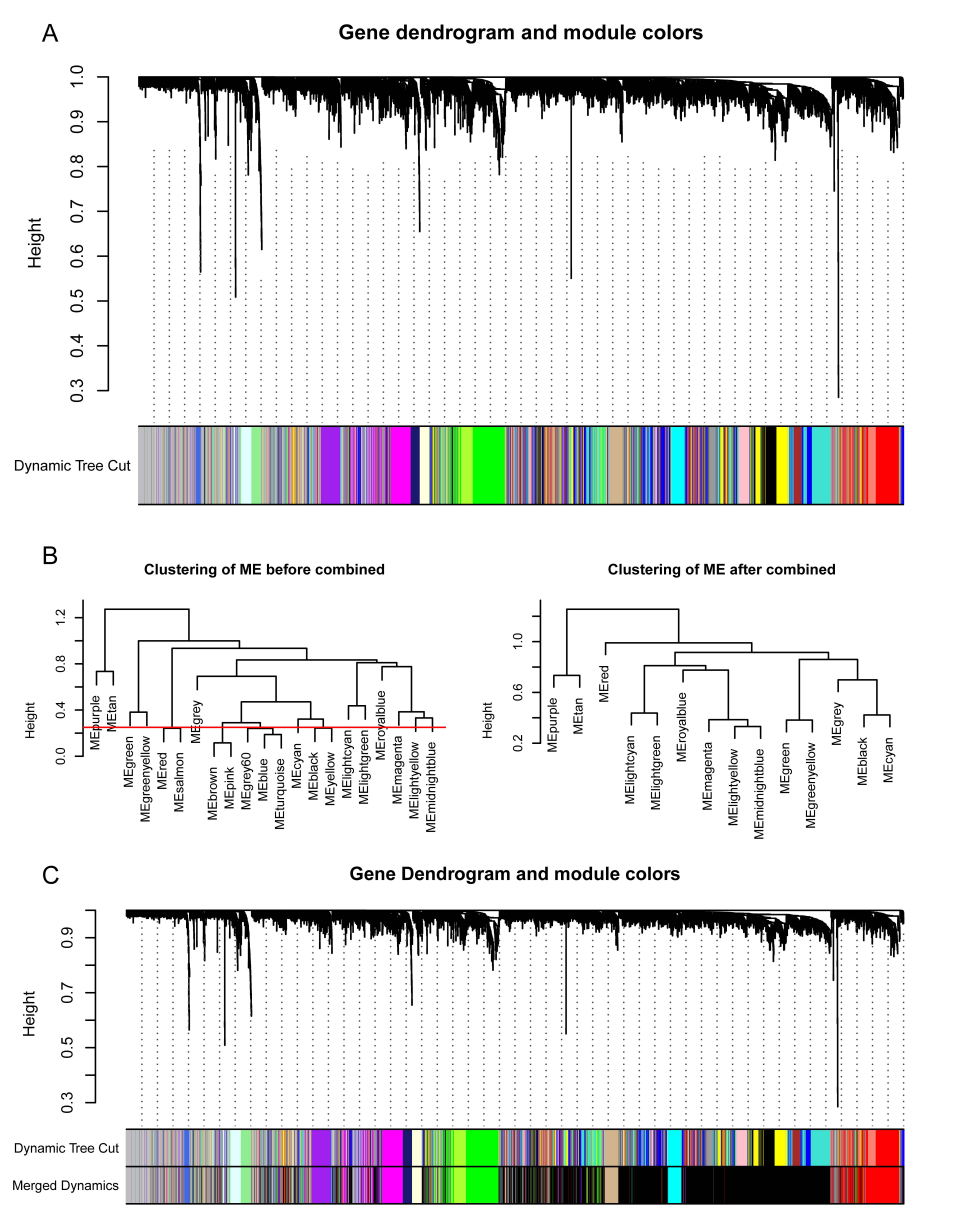
**Construction of weighted gene co-expression network of AF-VHD and SR-VHD samples**. (A) Gene dendrograms and modules were acquired using average linkage hierarchical clustering with dissimilarity according to topological overlap. Color beneath each row is a reflection of module assignment; there are different colors for different modules. (B) Dendrogram clustering was performed with 0.25 as height to identify similar modules. (C) After dynamic tree cutting and merging, 14 gene modules were obtained.

Two outliers (Sample15/GSM3182694; Sample26/GSM3182705) were observed (**Supplementary Fig. 1**) in the atrium samples in GSE115574. A total of 26 AF with VHD samples and 31 SR with VHD samples were included in the analysis after discarding the outliers. To satisfy the scale-free assumption of the constructed biological networks, the soft threshold power β = 9 was selected for the AF- and SR-VHD samples (**Supplementary Fig. 2A,B**). By calculating the scale-free topology fitting index, the value of $R^{2}$ was shown to reach 0.91. The results were represented via a histogram and a linear plot (**Supplementary Fig. 2C,D**). Additionally, an average hierarchical linkage clustering was calculated using TOM-based dissimilarity measurements with a minimum size of 30 genes. Modules of every gene cluster were identified based on the hierarchical cluster analysis (Fig. 4A). Following the merging of similar modules with a clustering height cut-off of 0.25 (Fig. 4B), a total of 14 modules with a high credibility were obtained, with the initial and merged modules presenting under the clustering tree (Fig. 4C).

### 3.5 Identification of the Most Significant Modules and Hub Genes

**Fig. 5.**
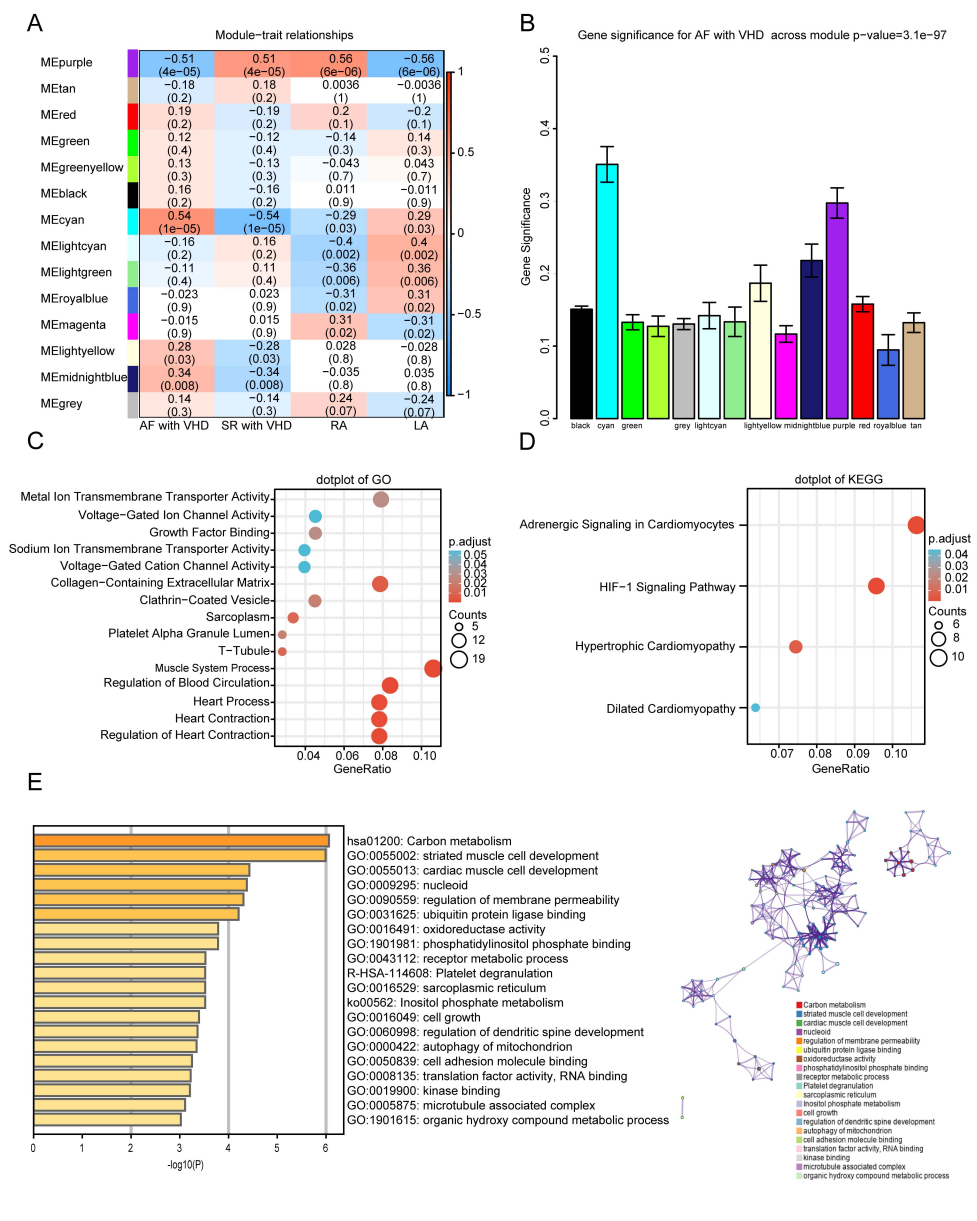
**Functional enrichment of the key module**. (A) Module-trait relationship plot. There is one row for each module eigengene and one column for each trait. Correlation coefficients and *p*-values are displayed in each cell. Red represents a positive correlation, and blue represents a negative correlation. RA, right atrium; LA, left atrium. (B) Bar plot of module significance (MS) defined as the average absolute value of gene significance (GS) for all genes in a module. The cyan and purple modules are the most promising. (C,D) GO enrichment and KEGG analyses for purple module. (C) Top five GO category terms of biological process (BP), cellular component (CC), and molecular function (MF) were identified. (D) Top four terms of KEGG analysis. A *p*-value (adjusted) < 0.05 was considered significant. (E) Top 20 clusters from Metascape GO enrichment analysis of cyan module-associated genes.

**Fig. 6.**
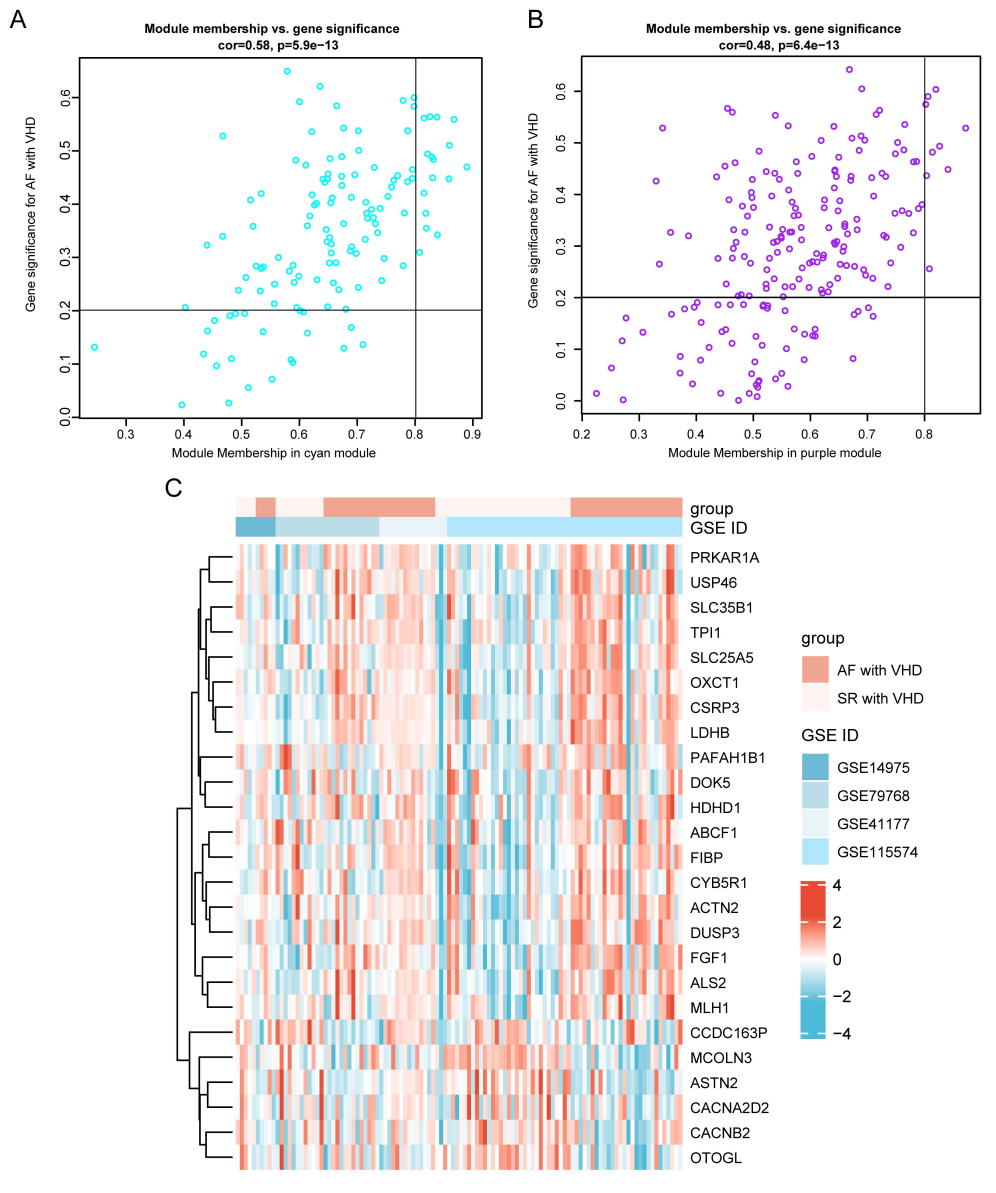
**Identification of hub genes in the key module**. (A) Scatterplot of disease status for gene significance (GS) vs. disease status for module membership (MM) in cyan module. (B) Scatterplot of GS vs. MM for purple module. Hub genes were screened based on the following criteria: GS >0.2 and MM >0.8. (C) Although the two groups of samples are not completely separated, heatmaps can show the expression level and trend of change patterns of hub genes in key modules.

The module-trait relationships illustrate the correlation between the available clinical features (disease status, tissue site) and each module in GSE115574 by calculating the value of MS (Fig. 5A). Notably, the ME of the cyan module (r = 0.54, *p* = 1 × ${\mathrm{10}}^{-5}$) showed the highest linkage with AF-VHD, followed by the purple module (r = –0.51, *p* = 4 × ${\mathrm{10}}^{-5}$). Additionally, the mean GS across all genes in each module was illustrated by the MS values displayed in a bar diagram. The cyan and purple modules showed to be of substantial interest (Fig. 5B). Therefore, the cyan and purple modules were selected as the main focus modules, and scatter plot analysis was conducted to determine the correlation between the GS and MM of the cyan (Fig. 6A) and purple (Fig. 6B) modules. Highly connected genes (hub genes) were defined using module connectivity (MM >0.8) and clinical trait relationship (GS >0.2). Under these criteria, 25 genes were identified as candidates for further analysis. MM and GS values were detailed in **Supplementary Table 3**. A heatmap visualizing gene expression changes for 25 hub genes in merged datasets was shown in Fig. 6C.

### 3.6 Function Enrichment Analysis of Genes in Key Modules

GO and KEGG analyses were performed to gain a deeper understanding of the biological functions of genes in the cyan and purple modules. The GO results showed that genes in the purple module were mainly clustered in MF, CC, and BP, including “metal ion transmembrane transporter activity”, “voltage-gated ion channel activity”, “sarcoplasm”, “T-tubule”, “muscle system process”, “regulation of blood circulation”, and “regulation of heart contraction” (Fig. 5C). In addition, KEGG analysis of genes in the purple modules showed that they were mainly enriched in the following terms: adrenergic signaling in cardiomyocytes, HIF-1 signaling pathway, hypertrophic cardiomyopathy, and dilated cardiomyopathy (Fig. 5D). The top five terms from the Metascape analysis of the cyan module included carbon metabolism, striated muscle cell development, cardiac muscle cell development, nucleoid, and regulation of membrane permeability. The top 20 cluster-enriched sets are shown in Fig. 5E.

### 3.7 Screening Candidate Hub Genes

**Fig. 7.**
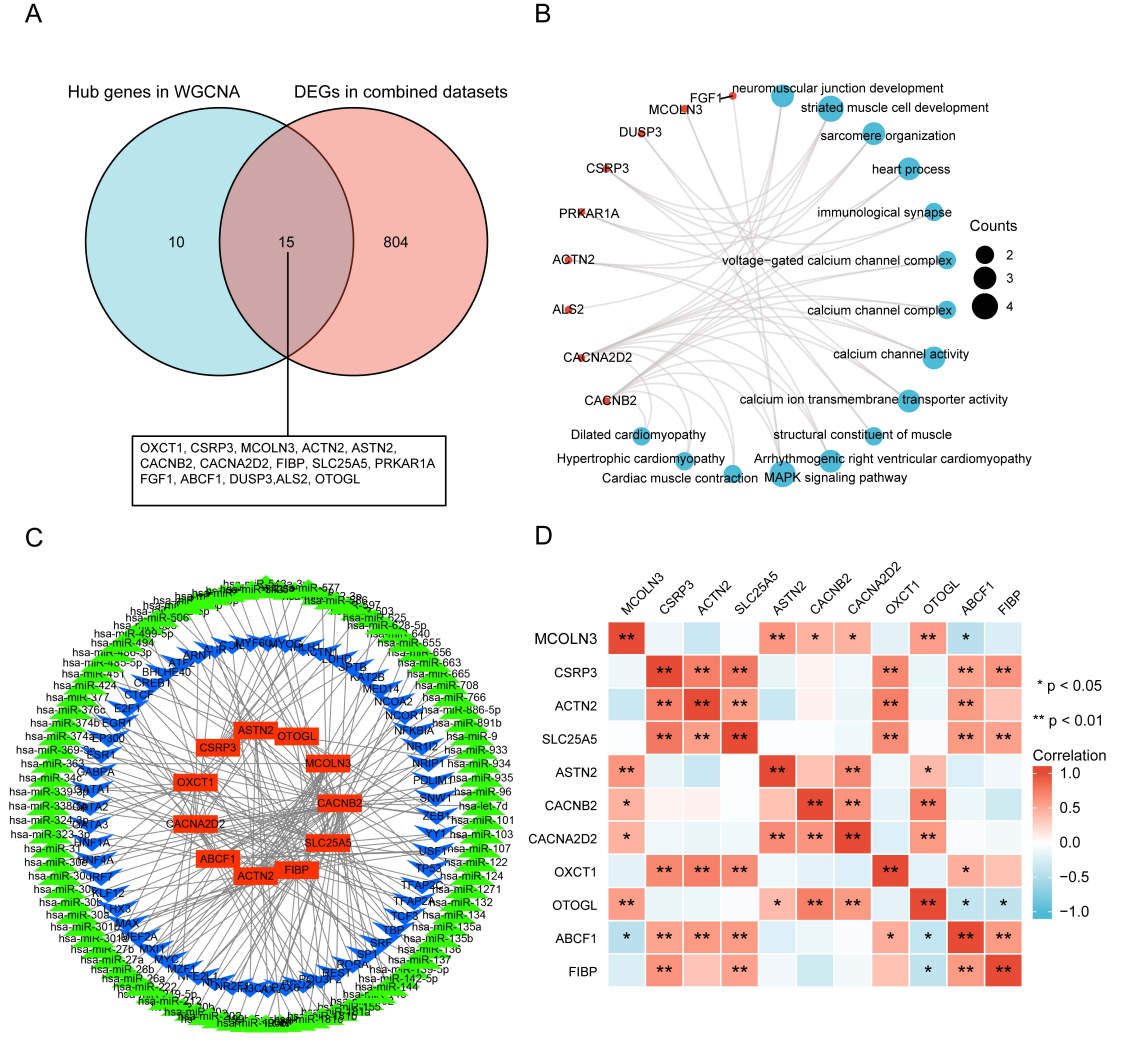
**Overlap of genes in key modules with DEGs and construction of TF-miRNA networks**. (A) Venn diagram represents unique and shared genes between common DEGs in merged datasets and hub genes in WGCNA. A total of 15 candidate hub genes were identified. (B) Functional enrichment analysis of candidate hub genes. (C) TF-miRNA regulatory network. Red rectangle represents hub genes, blue V represents TFs, and green triangle represents miRNAs. (D) Heatmap with Spearman’s correlations among 11 hub genes.

In order to screen the candidate hub genes for further analysis, DEGs of the combined datasets (GSE115574, GSE41177, GSE79768, and GSE41177) were overlapped with the hub genes in the key modules using a Venn diagram. A total of 15 candidate hub genes were identified (Fig. 7A). They were mainly enriched in the Mitogen-Activated Protein Kinase (MAPK) signaling pathway (gene ratio 4/10), arrhythmogenic right ventricular cardiomyopathy (gene ratio 3/10), dilated cardiomyopathy, cardiac muscle contraction (gene ratio 2/10), structural constituent of muscle, calcium channel activity (gene ratio 4/14), and calcium ion transmembrane transporter activity (gene ratio 3/14; Fig. 7B). To determine which TFs and miRNAs may be responsible for the altered candidate hub gene expression, transcription factor and miRNA analyses were performed using NetworkAnalyst. A total of 61 TFs and 96 miRNAs were identified (Fig. 7C). The correlation between 11 hub genes was determined using Spearman’s rank test. A positive correlation was observed between *CSRP3* and *SLC25A5* (Fig. 7D).

### 3.8 Identification of Real Hub Genes in AF-VHD

**Fig. 8.**
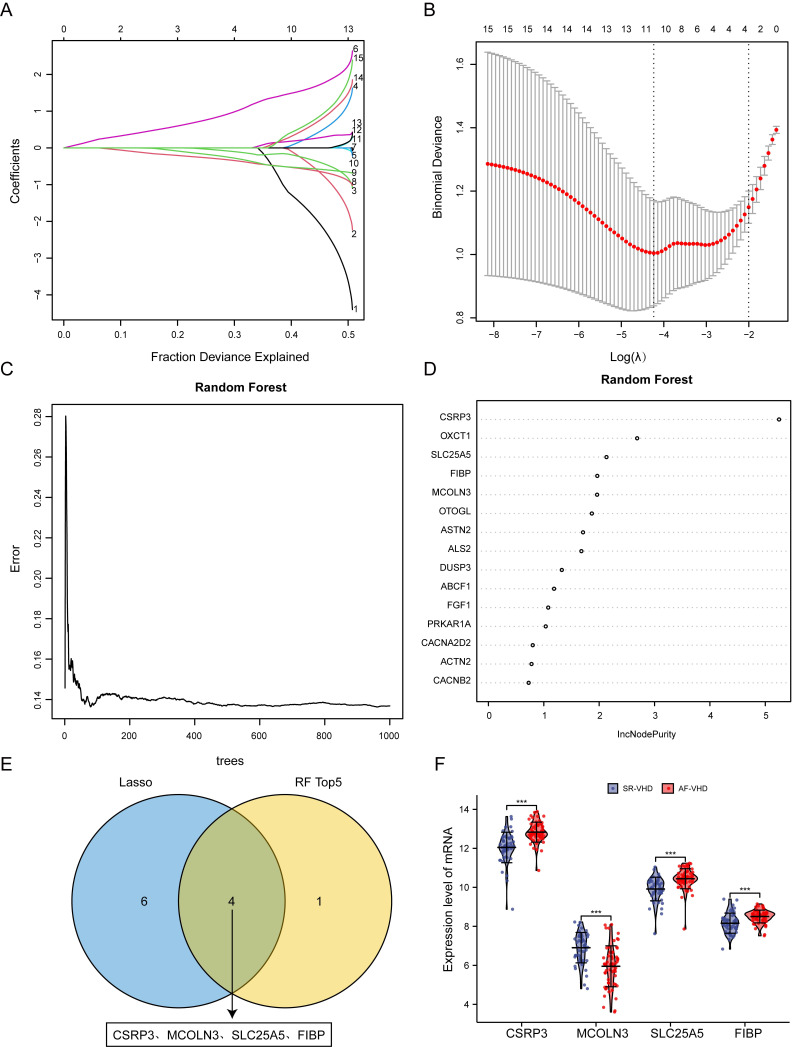
**Identification of real hub gene signatures using machine learning**. (A) Lasso coefficient profiles of the key genes in AF-VHD. Coefficients are illustrated via corresponding fraction deviance. (B) Selection of tuning parameter in Lasso regression models. This is a plot of the binomial deviance metrics (the y-axis) against log(λ) (the bottom x-axis). Red dot indicates average deviance values for each model with a given λ. (C) Error rate of the random forest model (1000 trees). (D) Variable importance measure plot with horizontal axis as IncNodePurity. (E) Venn diagram showing Lasso and RF sharing four common genes (*CSRP3*, *MCOLN3*, *SLC25A5*, and *FIBP*). (F) mRNA expression levels of *CSRP3*, *MCOLN3*, *SLC25A5*, and *FIBP* in AF-VHD and SR-VHD sample groups based on merged datasets.

Lasso and RF analyses were performed to screen signatures within 15 candidate hub genes in AF-VHD. First, the Lasso algorithm identified ten signatures—*CSRP3*, *MCOLN3*, *SLC25A5*, *FIBP*, *ABCF1*, *ACTN2*, *ASTN2*, *CACNA2D2*, *OTOGL*, and *DUSP3*—under the condition of the best penalty parameter (λ) (Fig. 8A,B). Using RF, the top five most important variables were screened, which were *CSRP3*, *OXCT1*, *SLC25A5*, *FIBP*, and *MCOLN3* (Fig. 8C,D). After intersecting the gene signatures selected by Lasso and RF, *CSRP3*, *MCOLN3*, *SLC25A5*, and *FIBP* were determined to be the real hub genes in AF-VHD (Fig. 8E). The mRNA expression levels of hub genes in SR- and AF-VHD were investigated (Fig. 8F).

### 3.9 Construction and Validation of Diagnosis Model 

**Fig. 9.**
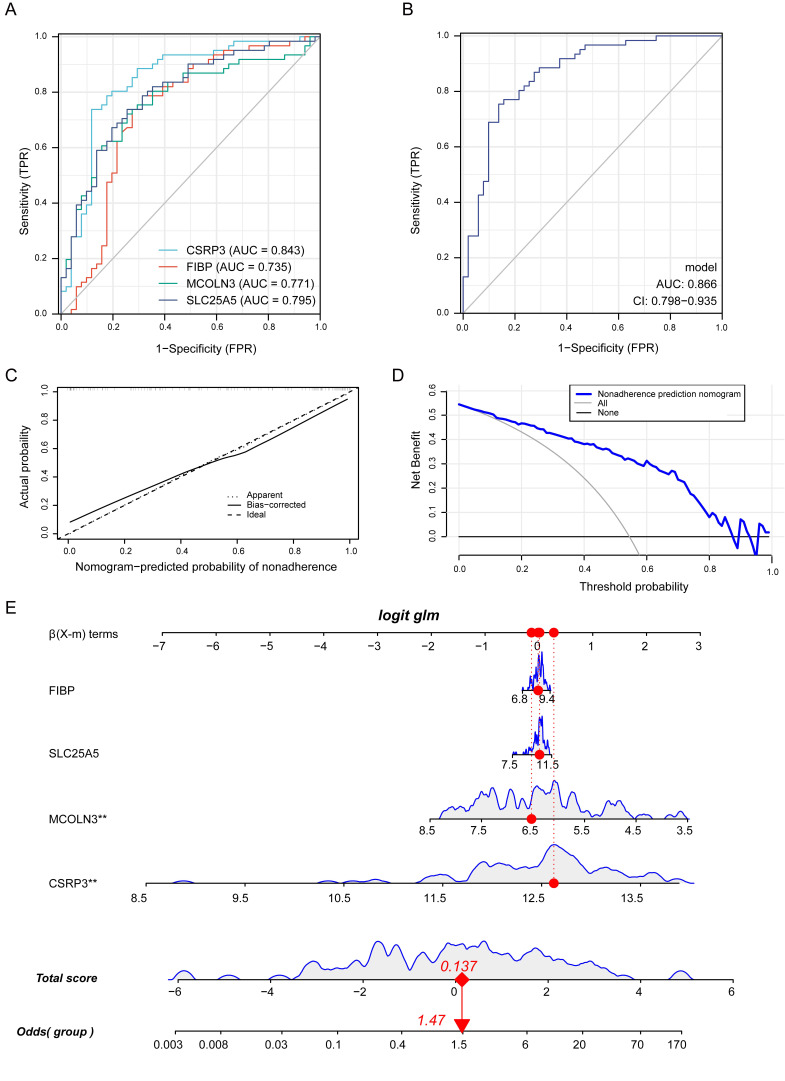
**Identification of crucial genes and evaluation of their clinical significance**. (A) ROC analysis for individual *MCOLN3*, *CSRP3*, *SLC25A5*, and *FIBP* genes in AF-VHD vs. SR-VHD in merged datasets. (B) Estimation of clinical diagnostic efficacy of four crucial gene signature (AUC = 0.866, 95% CI = 0798–0.935) (*Logistic regression model* = –20.0615 + 1.838 × *CSRP3* + –0.9575 × *MCOLN3* + 0.1821 × *SLC25A5* + 0.1969 × *FIBP*) (ROC, receiver-operating characteristic; AUC, area under curve). (C) Predictive ability of the diagnosis model represented by a calibration curve. (D) Decisions based on the diagnosis model had a better net benefit and broader threshold probability. (E) Construction of a nomogram model based on the four crucial genes. The potential clinic significance of *CSRP3* and *MCOLN3* were statistically significant.

The ROC analysis was conducted to further validate the diagnostic value of the hub genes in merged datasets. The results demonstrated that *CSRP3* (Area Under Curve, AUC 0.843), *MCOLN3* (AUC 0.771), *SLC25A5* (AUC 0.795), and *FIBP* (AUC 0.735) had a general ability to discriminate between AF with VHD and SR with VHD (Fig. 9A). Multiple biomarkers were combined as a sensitive screening index for AF-VHD in order to improve the diagnosis sensitivity. A combined diagnosis model of four vital genes was used to show that the AUC value of AF-VHD reached 0.866 (95% confidence interval (CI): 0.798–0.935; Fig. 9B). Calibration curves revealed that the diagnosis model had a good performance when predicting AF-VHD incidence (Fig. 9C). The blue line in the DCA curve remained above the gray and black lines between 0 and 0.8, implying that decisions based on the diagnosis model may be beneficial to AF-VHD patients (Fig. 9D). A nomogram was established using the RMS package for the diagnosis of AF-VHD based on the four crucial genes (*CSRP3*, *MCOLN3*, *SLC25A5* and *FIBP*) (Fig. 9E).

### 3.10 Key Genes Specifically Expressed in Heart Tissue

**Fig. 10.**
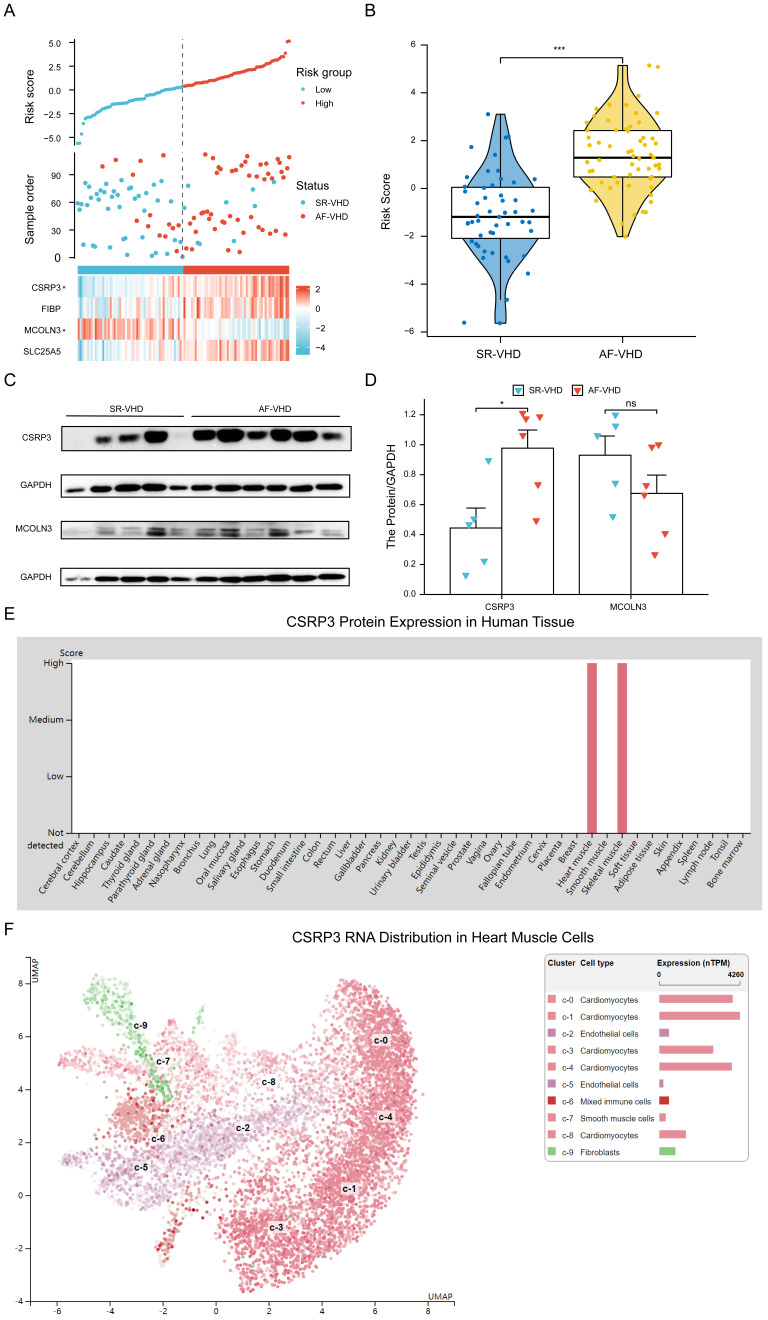
***CSRP3* is specifically expressed in the heart**. (A) Risk score distribution, disease status, and gene expression values of final predictors in the combined datasets. Dotted black line represents the median risk score cutoffs for classifying patients as high- or low-risk. Blue dots represent SR-VHD, and red dots represent AF-VHD. Heatmap showing gene expression values for corresponding sample. (B) Risk score differences between SR-VHD and AF-VHD. (C,D) *CSRP3* and *MCOLN3* protein expression levels were analyzed and quantified in atrial tissue from AF-VHD and SR-VHD patients. (E) In human tissue,* CSRP3* expression was predominantly found in heart and skeletal muscle. (F) The mRNA levels of *CSRP3* expression in different cell types showed that *CSRP3* was mainly expressed in cardiomyocytes.

Four genes were selected to calculate the risk score according to their coefficients, where risk score = –*20.0615 + 1.838*×* CSRP3 + –0.9575*×* MCOLN3 + 0.1821*×* SLC25A5 + 0.1969*×* FIBP.* Based on the median risk score, patients were categorized into high-risk and low-risk groups. As indicated in Fig. 10A, the prevalence of AF-VHD was significantly increased with an increased risk score in combined GSE datasets. The AF-VHD group had higher risk scores compared to the SR-VHD group (Fig. 10B). The *CSRP3* and *MCOLN3* protein expression levels in left atrial appendages from SR and AF patients with VHD were determined. Results indicated that the protein levels of *CSRP3* were higher in the AF-VHD samples than in the SR-VHD controls, whereas the *MCOLN3* expression did now show a significant difference in the two groups (Fig. 10C,D). The HPA tool showed that *CSRP3* was specifically expressed in the heart and skeletal muscle (Fig. 10E). The Single Cell Type Atlas in HPA indicated the expression of *CSRP3* in different cell types. All data were derived from the available published single-cell RNA sequencing analysis (Fig. 10F).

## 4. Discussion

AF is one of the most common tachyarrhythmias observed in the clinic. It increases patient morbidity and mortality, imposes an economic burden on patients, and seriously affects their quality of life [32]. VHD dramatically increases the risk of AF [33, 34]. Nevertheless, the mechanism for development of VHD into AF-VHD is still not completely understood. Therefore, it is essential to investigate the progression of AF-VHD and to identify specific biomarkers and potential therapeutic targets.

Based on previous studies, it has been shown that immune infiltration and atrial fibrosis are involved in the pathophysiological process of AF-VHD [8, 9, 10]. However, despite much work in this field, accurate and specific diagnostic biomarkers for AF-VHD are lacking. Our study identified four such biomarkers: *CSRP3*, *MCOLN3*, *SLC25A5*, and *FIBP*. *CSRP3* and *MCOLN3* in particular have important biological and clinical implications.

Microtubule-associated protein *CSRP3*, affiliated with the cysteine-rich protein (*CSRP/CRP*) family, is expressed in both cardiac and muscle tissue. *CSRP3* plays a pivotal role in the development and maintenance of cardiac cytoarchitectural organization [35, 36]. It was found to be differentially expressed in AF-VHD and potentially related to myocardial contractility [7]. Evidence has shown that *CSRP3* mutations can result in both hypertrophic cardiomyopathy (HCM) and dilated cardiomyopathy (DCM) in patients [34, 35]. HCM and DCM have been shown to be related to the occurrence and development of AF, which indirectly revealed that *CSRP3* might be connected to the development of AF-VHD. Li *et al*. [36] also demonstrated that cardiomyocytes derived from human embryonic stem cells with *CSRP3* deficiency mimic heart failure (HF) 30 days after differentiation, increasing reactive oxygen species generation and exhibiting mitochondrial damage and impaired ${\mathrm{Ca}}^{2+}$ handling. By restoring ${\mathrm{Ca}}^{2+}$ homeostasis, verapamil can trigger an inhibitory effect on HCM and HF, indicating that elevated intracellular ${\mathrm{Ca}}^{2+}$ concentration plays a critical role in the pathogenesis of *CSRP3* deficiency [36]. Our experimental results implied that *CSRP3* is highly expressed in AF-VHD, and the higher the *CSRP3* expression, the greater the odds of belonging to the AF-VHD group. Additionally, the enrichment analysis revealed that *CSRP3* is related to heart development. Single-cell analysis also showed that *CSRP3* is specifically expressed in myocardial cells. In conclusion, *CSRP3* was shown to be specifically highly expressed in AF-VHD, with a potential clinical significance in the diagnosis of AF-VHD, which may indicate that *CSRP3* is a potential biomarker, as well as a promising therapeutic target, for AF-VHD.

*SLC25A5*, also known as ANC3 or ANT2, is a member of the mitochondrial carrier subfamily of solute carrier protein genes. *SLC25A5* is highly expressed in high energetic demand organs, such as the heart, kidney, liver, and spleen, contributing to mitochondrial energy metabolism regulation and apoptosis prevention [37, 38]. However, the role of *SLC25A5* in AF is unknown. In our study, we found that *SLC25A5* is positively correlated with *CSRP3*, indicating that *SLC25A5* might also participate in the process of myocardial hypertrophy.

*MCOLN3*, also known as *TRPML3*, is a gene with the highest correlation with AF-VHD among the crucial genes. *MCOLN3/TRPML3* is a cation channel permeable to ${\mathrm{Ca}}^{2+}$ expressed in multiple subcellular compartments with dynamic localization. The findings by Kim *et al*. [39] has demonstrated that ${\mathrm{Ca}}^{2+}$ is released with a robust response when *MCOLN3*/*TRPML3 *is activated intracellularly. It has been previously shown that *MCOLN3* regulates autophagy by specifically interacting with mammalian GABA(A) Receptor-Associated Protein Like 1 (ATG8) homologue GATE16 [40]. Nevertheless, the presence of *MCOLN3* in the heart has been rarely reported. Recently, Düzen *et al*. [41] found that the expression of *MCOLN3* is up-regulated in patients with non-valvular AF (NVAF). It was the first study to reveal the expression pattern of a leukocyte TRP channel gene in NVAF. Our study indicated from the reverse side that the *MCOLN3/TRPML3* expression was reduced in valvular AF (VAF), which proved the expression of *MCOLN3* was associated with AF from another perspective. However, the results showed no difference in *MCOLN3* protein level between the AF-VHD and control groups. This might ybe due to an yinsufficient sample size in the study. In summary, *MCOLN3* plays an essential role in the regulation of ${\mathrm{Ca}}^{2+}$ trafficking, which may mediate the development of AF-VHD. However, the detailed action mechanism for *MCOLN3* in AF-VHD remains poorly understood, and further research is needed.

*FIBP* interacts directly with the fibroblast growth factor 1 (*FGF1*) [42]. *FIBP *is known to be involved in the FGF receptor signaling pathway and platelet aggregation [42, 43]. Although few studies have reported on the effect of *FIBP* on AF or VHD, Lu *et al*. [44] demonstrated that *FGF1* might be involved in AF via modification of oxidative stress and sodium/calcium homeostasis, suggesting that FIBP may genetically interact with *FGF1* to regulate the development of AF.

Our study has some limitations. First, due to the limited sample size derived from the public database, further research with a larger sample size should be conducted to strengthen the conclusion. Second, the biological and molecular functions of these molecules will need to be determined through further experimental studies.

## 5. Conclusions

In ysummary, four crucial genes (*CSRP3*, *MCOLN3*, *SLC25A5*, and *FIBP*) associated with development of AF-VHD were identified using comprehensive bioinformatics yanalysis. Based on their biological function and clinical value, these genes may be associated with the pathophysiological process of AF-VHD. These findings can facilitate the diagnosis and development of novel therapeutic targets for clinical disorders involving AF-VHD.

## Data Availability

The raw datasets were available from the GEO database (http://www.ncbi.nlm.nih.gov/geo/; GSE115574, GSE41177, GSE79768 and GSE14975).

## Supplementary Material

Supplementary material associated with this article can be found, in the online version, at https://doi.org/10.31083/j.rcm2307247.
