# Supplementary material for: Construction of Prediction Model for Atrial Fibrillation with Valvular Heart Disease Based on Machine Learning
Source: Rev Cardiovasc Med. 2022 Jun 28;23(7):247. doi: 10.31083/j.rcm2307247 (PMC11266776; doi:10.31083/j.rcm2307247)
Supplement: Supplementary file 1 [file 2153-8174-23-7-247-s1.docx]

Supplementary Table 1. GO enrichment of DEGs.

| Ontology | ID | Description | GeneRatio | BgRatio | *p*-value | p.adjust | q-value |
| --- | --- | --- | --- | --- | --- | --- | --- |
| BP | GO:0042119 | neutrophil activation | 44/704 | 498/18670 | 1.56e-07 | 4.82e-04 | 4.28e-04 |
| BP | GO:0002446 | neutrophil mediated immunity | 43/704 | 499/18670 | 4.29e-07 | 4.85e-04 | 4.30e-04 |
| BP | GO:0030198 | extracellular matrix organization | 33/704 | 368/18670 | 4.08e-06 | 0.002 | 0.002 |
| BP | GO:0030199 | collagen fibril organization | 11/704 | 54/18670 | 4.42e-06 | 0.002 | 0.002 |
| BP | GO:0043062 | extracellular structure organization | 35/704 | 422/18670 | 1.17e-05 | 0.005 | 0.004 |
| CC | GO:0062023 | collagen-containing extracellular matrix | 48/732 | 406/19717 | 1.33e-12 | 7.45e-10 | 6.21e-10 |
| CC | GO:0005788 | endoplasmic reticulum lumen | 33/732 | 309/19717 | 5.53e-08 | 1.55e-05 | 1.29e-05 |
| CC | GO:0005581 | collagen trimer | 16/732 | 87/19717 | 1.12e-07 | 2.09e-05 | 1.75e-05 |
| CC | GO:0005743 | mitochondrial inner membrane | 41/732 | 473/19717 | 4.68e-07 | 6.57e-05 | 5.47e-05 |
| CC | GO:0044420 | extracellular matrix component | 11/732 | 51/19717 | 2.10e-06 | 2.36e-04 | 1.96e-04 |
| MF | GO:0005201 | extracellular matrix structural constituent | 24/696 | 163/17697 | 2.45e-08 | 2.04e-05 | 1.91e-05 |
| MF | GO:0030020 | extracellular matrix structural constituent conferring tensile strength | 11/696 | 41/17697 | 3.46e-07 | 1.32e-04 | 1.23e-04 |
| MF | GO:0009055 | electron transfer activity | 18/696 | 114/17697 | 4.75e-07 | 1.32e-04 | 1.23e-04 |
| MF | GO:0019864 | IgG binding | 6/696 | 11/17697 | 1.41e-06 | 2.94e-04 | 2.75e-04 |
| MF | GO:0008201 | heparin binding | 21/696 | 169/17697 | 3.14e-06 | 5.22e-04 | 4.88e-04 |

BP, biological process; CC, cellular component; MF, molecular function.

Supplementary Table 2. KEGG enrichment of DEGs.

| Ontology | ID | Description | GeneRatio | BgRatio | *p*-value | p.adjust | q-value |
| --- | --- | --- | --- | --- | --- | --- | --- |
| KEGG | hsa04145 | Phagosome | 22/375 | 152/8076 | 1.76e-06 | 2.80e-04 | 2.45e-04 |
| KEGG | hsa04666 | Fc gamma R-mediated phagocytosis | 17/375 | 97/8076 | 1.88e-06 | 2.80e-04 | 2.45e-04 |
| KEGG | hsa04810 | Regulation of actin cytoskeleton | 23/375 | 218/8076 | 1.86e-04 | 0.014 | 0.012 |
| KEGG | hsa04933 | AGE-RAGE signaling pathway in diabetic complications | 13/375 | 100/8076 | 6.84e-04 | 0.034 | 0.030 |
| KEGG | hsa01200 | Carbon metabolism | 14/375 | 118/8076 | 0.001 | 0.046 | 0.040 |

Supplementary Table 3. Module membership and gene significance values of hub genes.

| Genes | GS | MM |
| --- | --- | --- |
| USP46 | 0.564046 | 0.825885 |
| LDHB | 0.563319 | 0.838149 |
| SLC25A5 | 0.56153 | 0.815656722 |
| CSRP3 | 0.559022 | 0.867484763 |
| FGF1 | 0.510443 | 0.859534331 |
| PRKAR1A | 0.494237 | 0.821950186 |
| FIBP | 0.48845 | 0.829482598 |
| DUSP3 | 0.483673 | 0.831751763 |
| OXCT1 | 0.469649 | 0.889841984 |
| PAFAH1B1 | 0.449342 | 0.830616563 |
| ALS2 | 0.447428 | 0.858771426 |
| TPI1 | 0.420867 | 0.815564424 |
| SLC35B1 | 0.405261 | 0.803830812 |
| CYB5R1 | 0.382893 | 0.818649545 |
| HDHD1 | 0.380336 | 0.822455861 |
| MLH1 | 0.370817 | 0.804113176 |
| ABCF1 | 0.35477 | 0.819541936 |
| ACTN2 | 0.342458 | 0.838711091 |
| DOK5 | 0.3093 | 0.808022526 |
| CACNA2D2 | -0.256 | 0.808576708 |
| MCOLN3 | -0.57459 | 0.802193565 |
| OTOGL | -0.60355 | 0.819929478 |
| ASTN2 | -0.48187 | 0.813877975 |
| CACNB2 | -0.43644 | 0.803692697 |
| CCDC163P | -0.41167 | 0.810645462 |

MM, module membership; GS, gene significance.


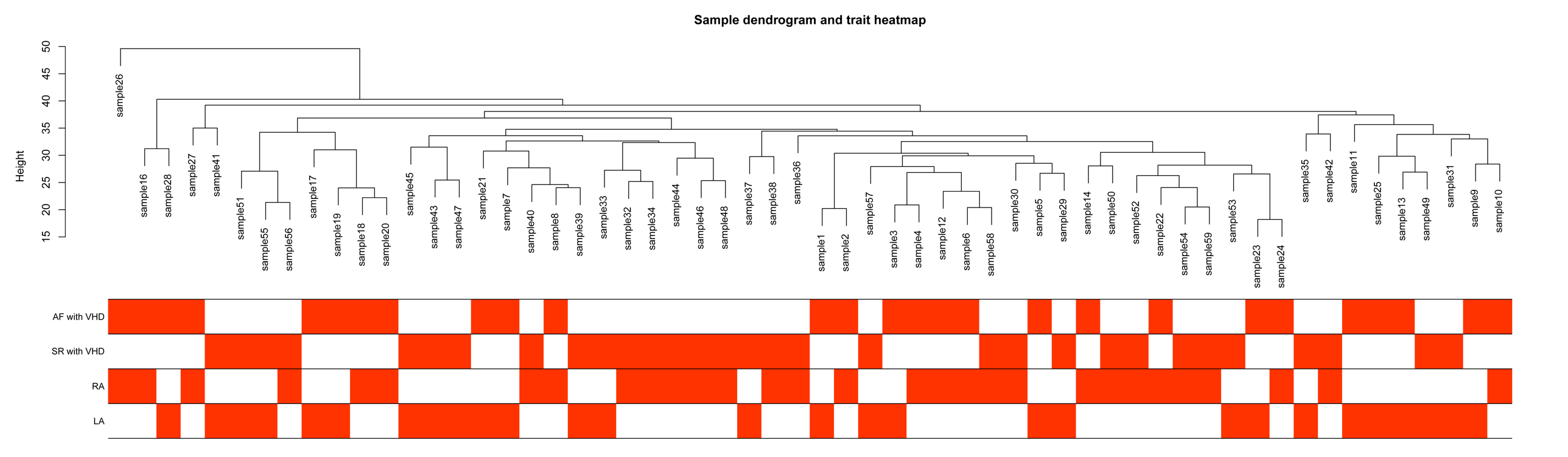


**Supplementary Fig. 1.** Sample dendrogram and trait heatmap.


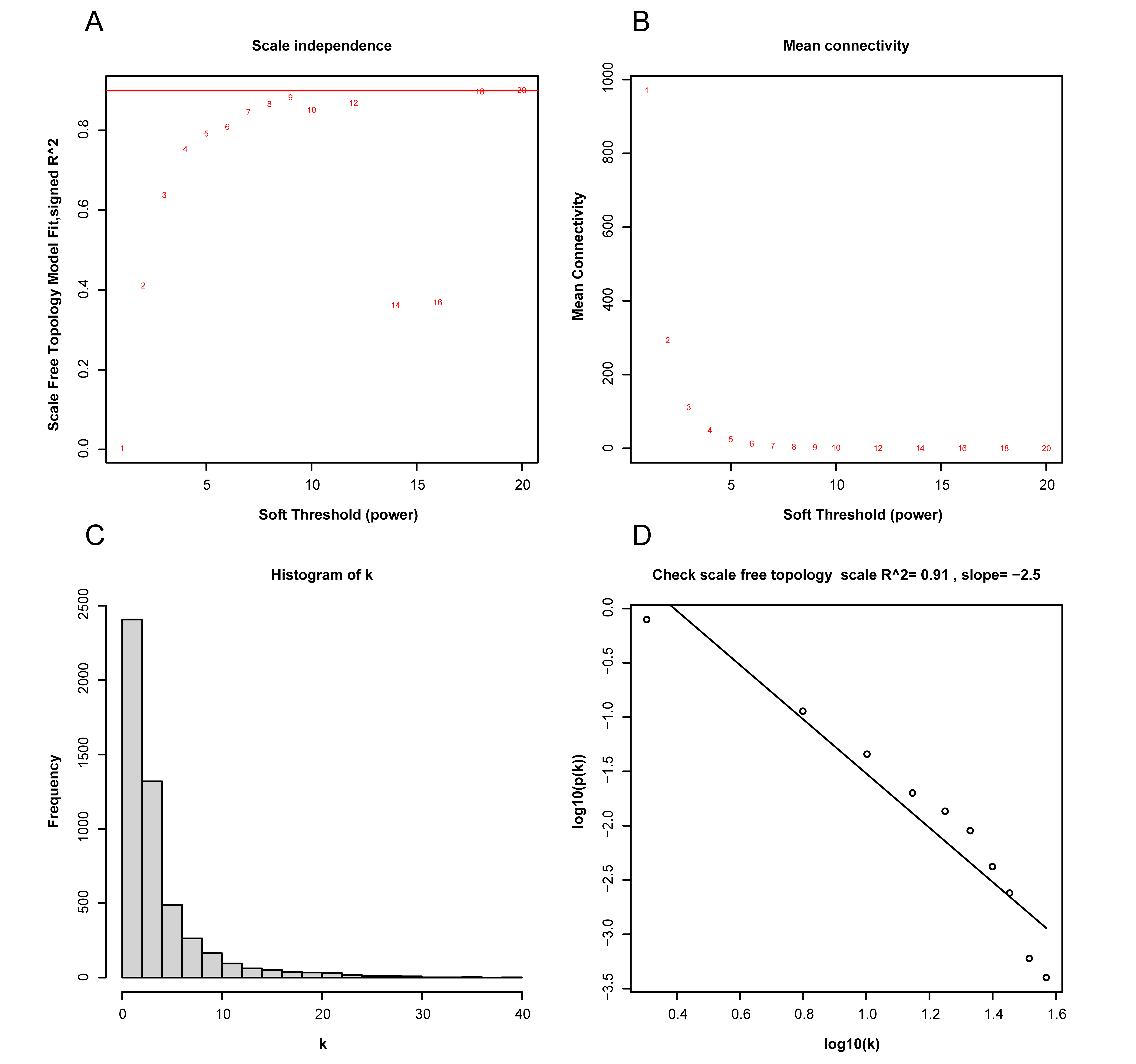


**Supplementary Fig. 2. Soft threshold filtering.** (A) Scale-free fit R^2^ vs. various soft thresholds. (B) Scale-free fit R^2^ vs. various soft thresholds mean connectivity. (C) Histogram of K. (D) Checking the scale free topology when β=9.
